# Finite state machine implementation for left ventricle modeling and control

**DOI:** 10.1186/s12938-019-0628-3

**Published:** 2019-01-30

**Authors:** Jacob M. King, Clint A. Bergeron, Charles E. Taylor

**Affiliations:** Department of Mechanical Engineering, University of Louisiana at Lafayette, 241 E. Lewis St. RM320, Lafayette, LA 70503 USA

**Keywords:** Left ventricular pressure–volume relationship, PV loop, Mock circulatory system, Cardiovascular lumped parameter modeling, Simulating cardiovascular hemodynamics, Patient-specific and population modeling

## Abstract

**Background:**

Simulation of a left ventricle has become a critical facet of evaluating therapies and operations that interact with cardiac performance. The ability to simulate a wide range of possible conditions, changes in cardiac performance, and production of nuisances at transition points enables evaluation of precision medicine concepts that are designed to function through this spectrum. Ventricle models have historically been based on biomechanical analysis, with model architectures constituted of continuous states and not conducive to deterministic processing. Producing a finite-state machine governance of a left ventricle model would enable a broad range of applications: physiological controller development, experimental left ventricle control, and high throughput simulations of left ventricle function.

**Methods:**

A method for simulating left ventricular pressure-volume control utilizing a preload, afterload, and contractility sensitive computational model is shown. This approach uses a logic-based conditional finite state machine based on the four pressure-volume phases that describe left ventricular function. This was executed with a physical system hydraulic model using MathWorks’ Simulink^®^ and Stateflow tools.

**Results:**

The approach developed is capable of simulating changes in preload, afterload, and contractility in time based on a patient’s preload analysis. Six pressure–volume loop simulations are presented to include a base-line, preload change only, afterload change only, contractility change only, a clinical control, and heart failure with normal ejection fraction. All simulations produced an error of less than 1 mmHg and 1 mL of the absolute difference between the desired and simulated pressure and volume set points. The acceptable performance of the fixed-timestep architecture in the finite state machine allows for deployment to deterministic systems, such as experimental systems for validation.

**Conclusions:**

The proposed approach allows for personalized data, revealed through an individualized clinical pressure–volume analysis, to be simulated in silico. The computational model architecture enables this control structure to be executed on deterministic systems that govern experimental left ventricles. This provides a mock circulatory system with the ability to investigate the pathophysiology for a specific individual by replicating the exact pressure–volume relationship defined by their left ventricular functionality; as well as perform predictive analysis regarding changes in preload, afterload, and contractility in time.

## Introduction

Every year since 1919, cardiovascular disease (CVD) accounted for more deaths than any other major cause of death in the United States [1]. Based on data collected by the National Health and Nutrition Examination Survey (NHANES), CVD was listed as the underlying cause of death in 30.8% of all deaths in 2014, accounting for approximately 1 of every 3 deaths in the U.S., while CVD attributed to 53.8% of all deaths in that year. Additionally, data accumulated from 2011 to 2014 revealed that approximately 92.1 million American adults currently have one or more types of CVD and that by 2030, projections estimate that 43.9% of the U.S. population will have some form of this disease.

Research has revealed that CVD is a leading contributor to Congestive Heart Failure (CHF) [2]. CHF is a medical condition that occurs when the heart is incapable of meeting the demands necessary for maintaining an adequate amount of blood flow to the body, resulting in ankle swelling, breathlessness, fatigue, and potentially death [2]. In 2012, the total cost for CHF alone was estimated to be $30.7 billion with 68% attributed to direct medical costs. Furthermore, predictions indicate that by 2030, the total cost of CHF will increase almost 127% to an estimated $69.7 billion [1]. This prediction is based on data that revealed that one-third of the U.S. adult population has the predisposing conditions for CHF. With research revealing that 50% of people who develop CHF will die within 5 years of being diagnosed [1, 3], the need to evaluate treatments for this widening patient population is of growing importance.

One treatment alternative for patients with late-stage CHF is the use of a ventricular assist device (VAD) to directly assist with the blood flow demands of the circulatory system [2]. Implantable VADs have proven their potential as a quickly implemented solution for bridge to recovery, bridge to transplant, and destination therapy [4]. Given the severity of CHF, and the impending need for supplemental support from these cardiac assist devices, effective methods of identifying the recipient cardiovascular profile and matching that to the operation of the VAD is critical to the success of the intervention.

The effectiveness of CHF diagnosis and treatment therapy depends on an accurate and early assessment of the underlying pathophysiology attributed to a specific type of CVD, typically by means of analyzing ventricular functionality [2, 5, 6]. Clinical application of non-invasive cardiac imaging in the management of CHF patients with systolic and/or diastolic dysfunction has become the standard with the use of procedures such as echocardiography [7–10]. Echocardiography is a non-invasive ultrasound procedure used to assess the heart’s structures and functionality, to include the left ventricular ejection fraction (LV_EF_), left ventricular end-diastolic volume (LV_EDV_), and left ventricular end-systolic volume (LV_ESV_). Three-dimensional echocardiography of adequate quality has been shown to improve the quantification of left ventricular (LV) volumes and LV_EF_, as well as provide data with better accuracy when compared with values obtained from cardiac magnetic resonance imaging [2, 11]. At present, echocardiography has been shown to be the most accessible technology capable of diagnosing diastolic dysfunction; therefore, a comprehensive echocardiography examination incorporating all relevant two-dimensional and Doppler data is recommended [2]. Doppler techniques allow for the calculation of hemodynamic variations, such as stroke volume (SV) and cardiac output (CO), based on the velocity time integral through the LV outflow tract area.

A left ventricular pressure–volume (LV-PV) analysis, employing hemodynamic principles, has effectively performed as a basis for understanding cardiac physiology and pathophysiology for decades [12, 13]. A LV-PV analysis has been primarily restricted to clinical investigations in a research environment; therefore, it has not been extensively used due to the invasive nature of the procedure [14, 15]. A broader predictive application for detecting and simulating CHF is more easily attainable with the development of single-beat methodologies that only rely on data collected through non-invasive techniques. These techniques include echocardiographic measurements of the left ventricular volume (LVV), Doppler data, the peripheral estimates of left ventricular pressure (LVP), and the timing of the cardiac cycle [16–21].

Utilizing data obtained non-invasively, population and patient-specific investigations can be conducted by simulating the LV-PV relationship obtained through the PV analysis by means of a mock circulatory system (MCS) [22, 23]. An MCS is a mechanical representation of the human circulatory system, essential for in vitro evaluation of VADs, as well as other cardiac assist technologies [24–29]. An MCS effectively simulates the circulatory system by replicating specific cardiovascular conditions, primarily pressure [mmHg] and flow rate [mL/s], in an integrated bench-top hydraulic circuit. Utilizing these hydraulic cardiovascular simulators and data obtained through a clinical PV analysis, the controls that govern the LV portion of the MCS could be driven to produce the PV relationship of: a CVD profile, specific population, or patient [30]. With research revealing the increasing need for these medical devices [31], a comprehensive in vitro analysis could be completed to assure a particular cardiac assist device treatment will be effective beforehand. The ability of an MCS to be able to replicate the exact PV relationship that defines the pathophysiology for a specific individual allows for a robust in vitro analysis to be completed, and a “patient specific diagnosis” created, ensuring a higher standard of patient care [32, 33].

The following is how this manuscript is presented. “Background” section summaries the principal theories governing the modeling of the PV relationship, its background in simulating cardiovascular hemodynamics within an MCS, and how a PV loop controller should perform for subsequent in vitro testing. “Method” section presents the proposed methodology for developing LV-PV control functionality is presented and utilizes a logic-based conditional finite state machine (FSM) and a physical system modeling approach, then the experimental results are presented in “Results” section. “Discussion” section concludes with a discussion regarding the results of this investigation, followed by “Conclusion” section which outlines the limitations of the approach and future investigations.

## Background

### Pressure–volume relationship

The efficacy of the PV relationship, often referred to as a PV loop, to describe and quantify the fundamental mechanical properties of the LV was first demonstrated in 1895 by Otto Frank [34]. Frank represented the cardiac cycle of ventricular contraction as a loop on a plane defined by ventricular pressure on the vertical axis and ventricular volume on the horizontal. By late twentieth century, the PV analysis was considered the gold standard for assessing ventricular properties, primarily due to the researched conducted by Suga and Sagawa [35–37]. Yet, this approach has failed to become the clinical standard for evaluating LV functionality due to the invasive nature of the procedure [14, 15]. However, due to recent advances single-beat methodologies, the practical application for PV analysis is expanding [18–20]. Most recently are the efforts published in 2018 by Davidson et al. with regard to the development of a beat-by-beat method for estimating the left ventricular PV relationship using inputs that are clinically accessible in an intensive care unit (ICU) setting and are supported by a brief echocardiograph evaluation [20].

**Fig. 1:**
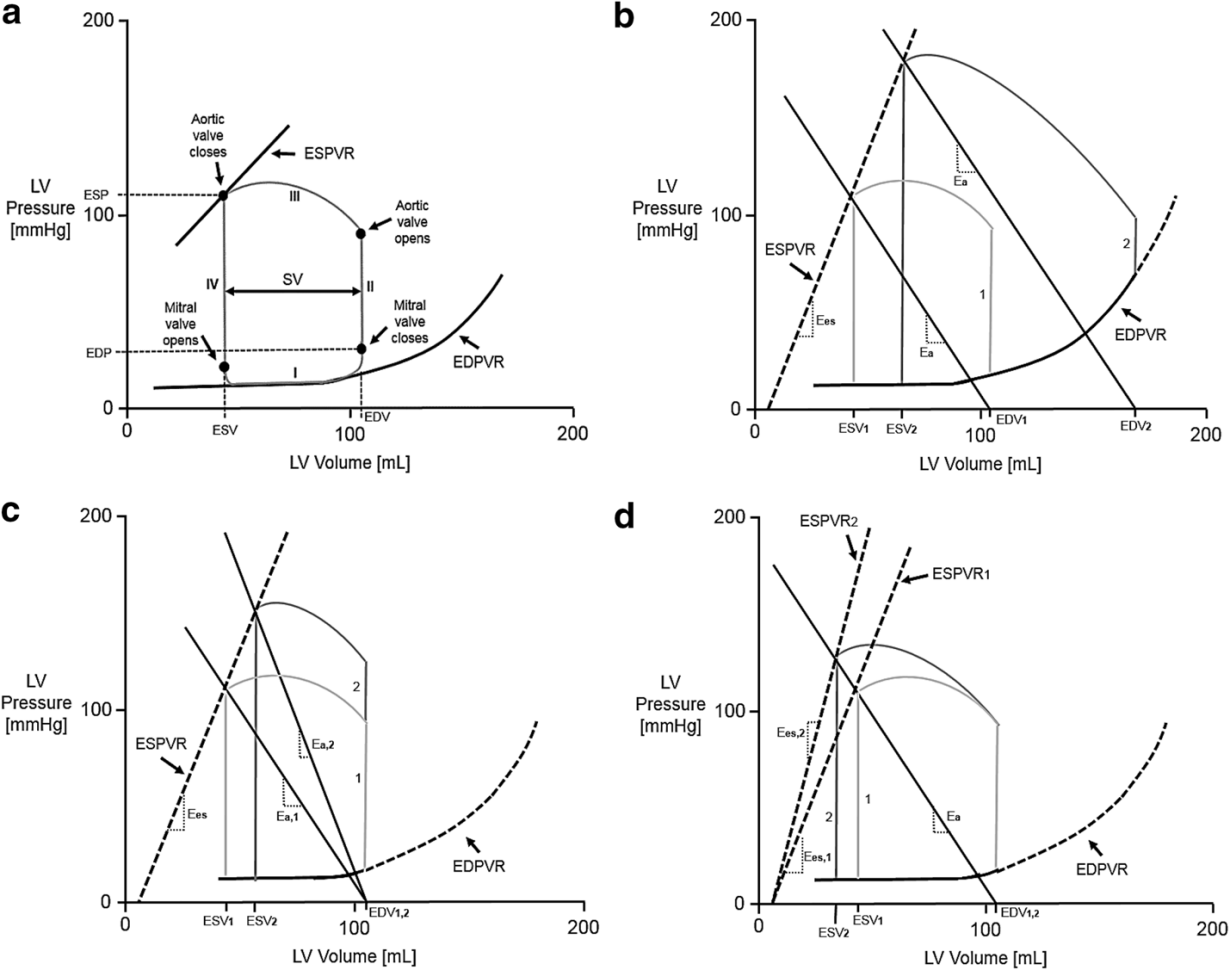
Left Ventricular Pressure–Volume Relationship (Stouffer [30]). **a** Schematic of LV pressure–volume loop in a normal heart. In Phase I, preceding the opening of the mitral valve, ventricular filling occurs with only a small increase in pressure and a large increase in volume, guided along the EDPVR curve. Phase II constitutes the first segment of systole called isovolumetric contraction. Phase III begins with the opening of the aortic valve; ejection initiates and LV volume falls as LV pressure continues to increase. Isovolumetric relaxation begins after the closure of the aortic valve constituting Phase IV. **b** Effects of increasing preload on a LV-PV loop with afterload and contractility remaining constant. Loop 2 has an increased preload compared to loop 1 by rolling the arterial elastance (E_a_) line parallel while keeping the slope (E_a_) constant, resulting in an increase in SV. **c** Effects of increasing afterload on a LV-PV loop with preload and contractility held constant. This consists of increasing the slope of the E_a_ line. **d** Effects of increasing contractility on a LV-PV loop with preload and afterload remaining constant. This consists of increasing the slope (E_es_) of the ESPVR line. Note that in **b**, **c**, and **d**, loop 2 represents the increase in the respective principle factor, i.e. preload, afterload, and contractility, when compared to loop 1

There has been extensive clinical and computational research into understanding the PV relationship, which is presented in Fig. 1 [12, 21, 30, 38]. However, for the purpose of repeatability within a MCS, the culmination of this knowledge can be summarized by simplifying the performance of the LV through three principal factors: preload, afterload, and contractility [24, 25]. These have significant implications on VAD performance [39].

A schematic of the LV pressure–volume loop in a normal heart is presented in Fig. 1a. In Phase I, ventricular filling occurs with only a small increase in pressure and a large increase in volume, guided along the EDPVR curve. Phase I can additionally be divided in two sub-phases, rapid filling governed by elastance of the ventricle and atrial systole that brings the ventricle into optimal preload for contraction. Phase II constitutes the first segment of systole called isovolumetric contraction. Phase III begins with the opening of the aortic valve; ejection initiates and LV volume falls as LV pressure continues to increase. Phase III can be divided into two sub-phases: rapid ejection and reduced ejection. Isovolumetric relaxation begins after the closure of the aortic valve constituting Phase IV.

Ventricular preload refers to the amount of passive tension or stretch exerted on the ventricular walls (i.e. intraventricular pressure) just prior to the systolic contraction [14, 29]. This load determines the end-diastolic sarcomere length and thus the force of contraction. Because the true sarcomere length is not easily measured clinically, preload is typically measured by ventricular pressure and volume at the point immediately preceding isometric ventricular contraction. This correlation is described through the end-systolic pressure–volume relationship (ESPVR); as well as through the end-diastolic pressure–volume relationship (EDPVR). The effects of increasing preload on the PV relationship is displayed in Fig. 1b; reduced isovolumetric contraction period and increased stroke volume.

Afterload is defined as the forces opposing ventricular ejection [14]. Effective arterial elastance (E_a_) is a lumped measure of total arterial load that incorporates the mean resistance with the pulsatile factors that vary directly with heart rate, systemic vascular resistance, and relates inversely with total arterial compliance. E_a_ is directly defined as the ratio of left ventricular end-systolic pressure (LV_ESP_) to SV. In practice, another measure of afterload is the LV_ESP_ at the moment ventricular pressure begins to decrease to less than systemic arterial pressure. The effects of increasing afterload are presented in Fig. 1c; increase in peak systolic pressure and decrease in stroke volume.

A acceptable clinical index of contractility that is independent of preload and afterload has not been completely defined [29]. In non-pathological conditions, contractility is best described by the pressure–volume point when the aortic valve closes. Contractility is typically measured by the slope of the ESPVR line, known as E_es_, which is calculated as $\frac{{\Delta {\text{P}}}}{{\Delta {\text{V }}}}$ [38]. An additional index of contractility is dP/dt_max_ which is the derivative of the maximum rate of ventricular pressure rise during the isovolumetric period. The effects of increasing contractility on the PV relationship is revealed in Fig. 1d; revealing the ability for the stroke volume to accommodate with increasing peak systolic pressure.

For a given ventricular state, there is not just a single Frank-Starling curve, rather there is a set or family of curves [29]. Each curve is determined by the driving conditions of preload, afterload, and inotropic state (contractility) of the heart. While deviations in venous return can cause a ventricle to move along a single Frank-Starling curve, changes in the driving conditions can cause the PV relationship of the heart to shift to a different Frank-Starling curve. This allows clinicians to diagnose the pathophysiological state of a dysfunctional heart by analyzing the PV relationship of a patient.

Additionally, it provides the ability to simulate diseased states: heart failure [14], valvular disease [29], or specific cardiovascular dysfunction seen in pediatric heart failure [40].

### Pressure–volume loop computational modeling

Comprehensive computationally modeling of the LV-PV relationship has been effectively reported since the mid-1980s, following the extensive work completed by Suga and Sagawa [34–36]. In 1986, Burkhoff and Sagawa first developed a comprehensive analytical model for predicting ventricular efficiency utilizing Windkessel modeling techniques and an understanding of the PV relationship principles previously developed by Suga and Sagawa. With the advancement and routine use of innovative technologies in the early twenty-first century (e.g. conductance catheter, echocardiography), there was a significant increase in research efforts to determine the potential clinical applications [12–15], improving predictive strategies [16–19], and refining computational models [41–43].

An elastance-based control of an electrical circuit analogue of a closed circulatory system with VAD assistance was developed in 2009 by Yu et al. [42]. Their state-feedback controller was designed to drive a voice coil actuator to track a reference volume, and consequently generate the desired ventricular pressure by means of position and velocity feedbacks. The controller was tested in silico by modifying the load conditions as well as contractility to produce an accurate preload response of the system. The MCS analogue and controller architecture was able to reproduce human circulatory functionality ranging from healthy to unhealthy conditions. Additionally, the MCS control system developed was able to simulate the cardiac functionality during VAD support.

In 2007, Colacino et al. developed a pneumatically-driven mock left ventricle as well as a native left ventricle model and connected each model to a numerical analogue of a closed circulatory system comprised of systemic circulation, a left atrium, and inlet/outlet ventricular valves [43]. The purpose of their research was to investigate the difference between preload and afterload sensitivity of a pneumatic ventricle, when used as a fluid actuator in a MCS, when compared to elastance-based ventricle computational model. Their research concluded that the elastance-based model performed more realistically when reproducing specific cardiovascular scenarios and that many MCS designs could be considered inadequate, if careful consideration is not made to the pumping action of the ventricle. Subsequent in vitro testing utilizing this control approach successfully reproduced an elastance mechanism of a natural ventricle by mimicking preload and afterload sensitivity [25]. Preload was modified by means of manually changing the fluid content of the closed loop hydraulic circuit, while afterload was varied by increasing or decreasing the systemic arterial resistance within a modified Windkessel model.

### Recent advancements in contractility-based control

An MCS simulates the circulatory system by accurately and precisely replicating specific cardiovascular hemodynamic variables, mainly the respective pressure (mmHg) and flow rate (mL/s) for key circulatory constituents, in an integrated bench-top hydraulic circuit [23]. While this human circulatory system model is not an all-inclusive replacement for an in vivo analysis of a cardiac assist device’s design, it is an effective method of evaluating fundamental design decisions beforehand by determining its influence on a patient’s circulatory hemodynamics in a safe and controlled environment. Published research efforts typically either involve the development of the system [22, 25, 26, 44–46] or the dissemination of the results of a particular in vitro investigation [27, 28].

In 2017, Wang et al. was able to replicate the PV relationship with controllable ESPVR and EDPRV curves on a personalized MCS based on an elastance function for use in the evaluation of VADs [21]. The numerical elastance models were scaled to change the slopes of the ESPVR and EDPVR curves to simulate systolic and diastolic dysfunction. The results of their investigation produced experimental PV loops that are consistent with the respective theoretical loop; however, their model only includes a means of controlling preload and contractility with no afterload control. Their model assumes afterload remains constant regardless of preload changes; due to the Frank-Starling mechanism, the ventricle reached the same LV_ESV_ despite an increase in LV_EDV_ and preload.

Jansen-Park et al., 2015, determined the interactive effects between a simulated patient with VAD assistance on an auto-regulated MCS which includes a means of producing the Frank-Starling response and baroreflex [24]. In their study, a preload sensitive MCS was developed to investigate the interaction between the left ventricle and a VAD. Their design was able to simulate the physiological PV relationship for different conditions of preload, afterload, ventricular contractility, and heart rate. The Frank-Starling mechanism (preload sensitivity) was modeled by regulating the stroke volume based on the measured mean diastolic left atrial pressure, afterload was controlled by modifying systemic vascular resistance by means of an electrically controlled proportional valve, and contractility was changed depending on the end diastolic volume. The effects of contractility, afterload, and heart rate on stroke volume were implemented by means of two interpolating three-dimensional look-up tables based on experimental data for each state of the system. The structure of their MCS was based on the design developed by Timms et al. [27]. The results of their investigation revealed a high correlation to published clinical literature.

In 2011, Gregory et al. was able to replicate a non-linear Frank-Starling response in a MCS by modifying preload by means of opening a hydraulic valve attached to the systemic venous chamber [44]. Their research was able to successfully alter left and right ventricular contractility by changing preload to simulate the conditions of mild and severe biventricular heart failure. The EDV offset and a sensitivity gain were manually adjusted through trial and error to produce an appropriate degree of contractility with a fixed ventricular preload. The shape of the ESPVR curve was then modified by decreasing MCS volume until the ventricular volumes approached zero. These efforts, validated using published literature, improved a previously established MCS design developed by Timms et al. [28].

These control architectures were primarily hardware determined, rather than software-driven. In some cases, reproducibility is inhibited due to the tuning of hemodynamic conditions by manually adjusting parameters until a desired response is achieved. Utilizing a conditional logic-based conditional finite state machine (FSM) and physical system modeling control approach, a software-driven controller could be developed to respond to explicitly-defined preload, afterload, and contractility events. This would enable the regulation of the PV relationship within an MCS’s LV section, without the limitation of dedicated hardware.

### Logic-based finite state machine (FSM) and physical system modeling tools

MathWorks’ Simulink^®^ is a model based design tool utilized for multi-domain physical system simulation and model-based design [47]. Simulink^®^ provides a graphical user interface, an assortment of solver options, and an extensive block library for accurately modeling dynamic system performance. Stateflow^®^ is a toolbox found within Simulink^®^ for constructing combinatorial and sequential decision-based control logic represented in state machine and flow chart structure. Stateflow^®^ offers the ability to create graphical and tabular representations, such as state transition diagrams and truth tables, which can be used to model how a system reacts to time-based conditions and events, as well as an external signal. The Simscape™ toolbox, utilized within the Simulink^®^ environment, provides the ability to create models of physical systems that integrate block diagrams acknowledged by real-world physical connections. Dynamic models of complex systems, such as those with hydraulic and pneumatic actuation, can be generated and controlled by assembling fundamental components into a schematic-based modeling diagram. An additional toolbox that was utilized in this approach was the Simscape Fluids™ toolbox which provides component libraries for modeling and simulating fluid systems. The block library for this toolbox includes all the necessary modules to create systems with a variety of domain elements, such as hydraulic pumps, fluid reservoirs, valves, and pipes. The advantage of using these toolbox libraries is that the blocks are version controlled and conformal to regulatory processes that mandate tractable computational modeling tools.

## Method

### Overview of methodology and model architecture

A method for simulating LV-PV control functionality utilizing explicitly defined preload, afterload, and contractility is needed for cardiovascular intervention assessment. The resulting solution must be capable of being compiled for hardware control of an MCS; deterministic processing compatible logic and architecture that would enable runtime setpoint changes. The approach used was a logic-based conditional finite state machine (FSM) based on the four PV phases that describe left ventricular functionality developed with a physical system hydraulic plant model using Simulink^®^. The proposed aggregate model consists of three subsystems to include: a preload/afterload/contractility-based setpoint calculator (“PV loop critical point determination” section), a FSM controller (“PV loop modeling utilizing a state machine control architecture approach” section), and a hydraulic testing system (“Hydraulic testing model utilizing MathWorks’ Simulink® and SimscapeTM toolbox” section). The last subsystem acts as the simulated plant to evaluate the control architecture that is formed by the first two subsystems. The proposed method allows for multiple uses which include the simulation of parameter effects in time and the simulation of personalized data, revealed through an individualized clinical PV analysis. This method provides the means to be simulated in silico and can be subsequently compiled for control of in vitro investigations. This provides an MCS with the ability to investigate the pathophysiology for a specific individual by replicating the exact PV relationship defined by their left ventricular functionality; as well as perform predictive analysis regarding changes in preload, afterload, and contractility with time. Of critical importance were the non-isovolumetric state behavior: non-linear EDPVR curve, rate-limited ejection, and energy-driven model of contraction. This investigation was developed utilizing Matlab R2017b and a Dell T7500 Precision workstation with 8.0 gigabytes of RAM, a Dual Core Xeon E5606 processor, and a Windows 7 64-bit operating system.

### PV loop critical point determination

A preload, afterload, and contractility sensitive computational model was developed utilizing Simulink^®^ for determining critical points for switching between PV loop states; the four phases described in Fig. 1. These critical points are LV End-Systolic Pressure (LV_ESP_), LV End-Systolic Volume (LV_ESV_), LV End-Diastolic Pressure (LV_EDP_), LV End-Diastolic Volume (LV_EDV_), LV End-Isovolumetric Relaxation Pressure (LV_EIRP_), LV End-Isovolumetric Relaxation Volume (LV_EIRV_), LV End-Isovolumetric Contraction Pressure (LV_EICP_), and LV End-Isovolumetric Contraction Volume (LV_EICV_). These can be resolved by the three equations that describe ESPVR, EDPVR, and E_a_. ESPVR is typically described as a linear equation with a positive slope (E_es_) and a negative or positive y-intercept, EDPVR can be defined with a third-order polynomial, while E_a_ is also linear and has a negative slope with a positive y-intercept [13]. Eqs. 1, 2, and 3 define the system of equations used to produce the critical points, where ESPVR, EDPVR, and E_a_ are Eqs. 1, 2, and 3 respectively.

1 $$P_{A} = a_{1} V_{A} + a_{0}$$

2 $$P_{B} = b_{3} V_{B}^{3} + b_{2} V_{B}^{2} + b_{1} V_{B} + b_{0}$$

3 $$P_{C} = c_{1} V_{C} + c_{0}$$

The point where Eqs. 1 and 3 intercept is LV_ESV_ and LV_ESP_ and solving produces Eqs. 4 and 5.

4 $$LV_{ESV} = \frac{{c_{0} - a_{0} }}{{a_{1} - c_{1} }}$$

5 $$LV_{ESP} = a_{1} \left( {\frac{{c_{0} - a_{0} }}{{a_{1} - c_{1} }}} \right) + a_{0}$$

Setting Eq. 3 equal to zero yields LV_EDV_, producing Eq. 6.

6 $$LV_{EDV} = \frac{{ - c_{0} }}{{c_{1} }}$$

Substituting Eq. 6 into Eq. 2 produces LV_EDP_.

7 $$LV_{EDP} = b_{3} \left( {\frac{{ - c_{0} }}{{c_{1} }}} \right)^{3} + b_{2} \left( {\frac{{ - c_{0} }}{{c_{1} }}} \right)^{2} + b_{1} \left( {\frac{{ - c_{0} }}{{c_{1} }}} \right) + b_{0}$$

Due to isovolumetric relaxation,

8 $$LV_{EIRV} = LV_{ESV}$$

Thus, substituting Eq. 4 into Eq. 2 yields Eq. 8 for LV_EIRP_.

9 $$LV_{EIRP} = b_{3} \left( {\frac{{c_{0} - a_{0} }}{{a_{1} - c_{1} }}} \right)^{3} + b_{2} \left( {\frac{{c_{0} - a_{0} }}{{a_{1} - c_{1} }}} \right)^{2} + b_{1} \left( {\frac{{c_{0} - a_{0} }}{{a_{1} - c_{1} }}} \right) + b_{0}$$

Lastly, due to isovolumetric contraction, LV_EICV_ equals LV_EDV_. The final unknown variable value to complete the four-phase cycle is LV_EICP_. This is resolved by utilizing an offset value based on LV_ESP_.

10 $$LV_{EICV} = LV_{EDV}$$

11 $$LV_{EICP} = LV_{ESP} - offset$$

**Fig. 2:**
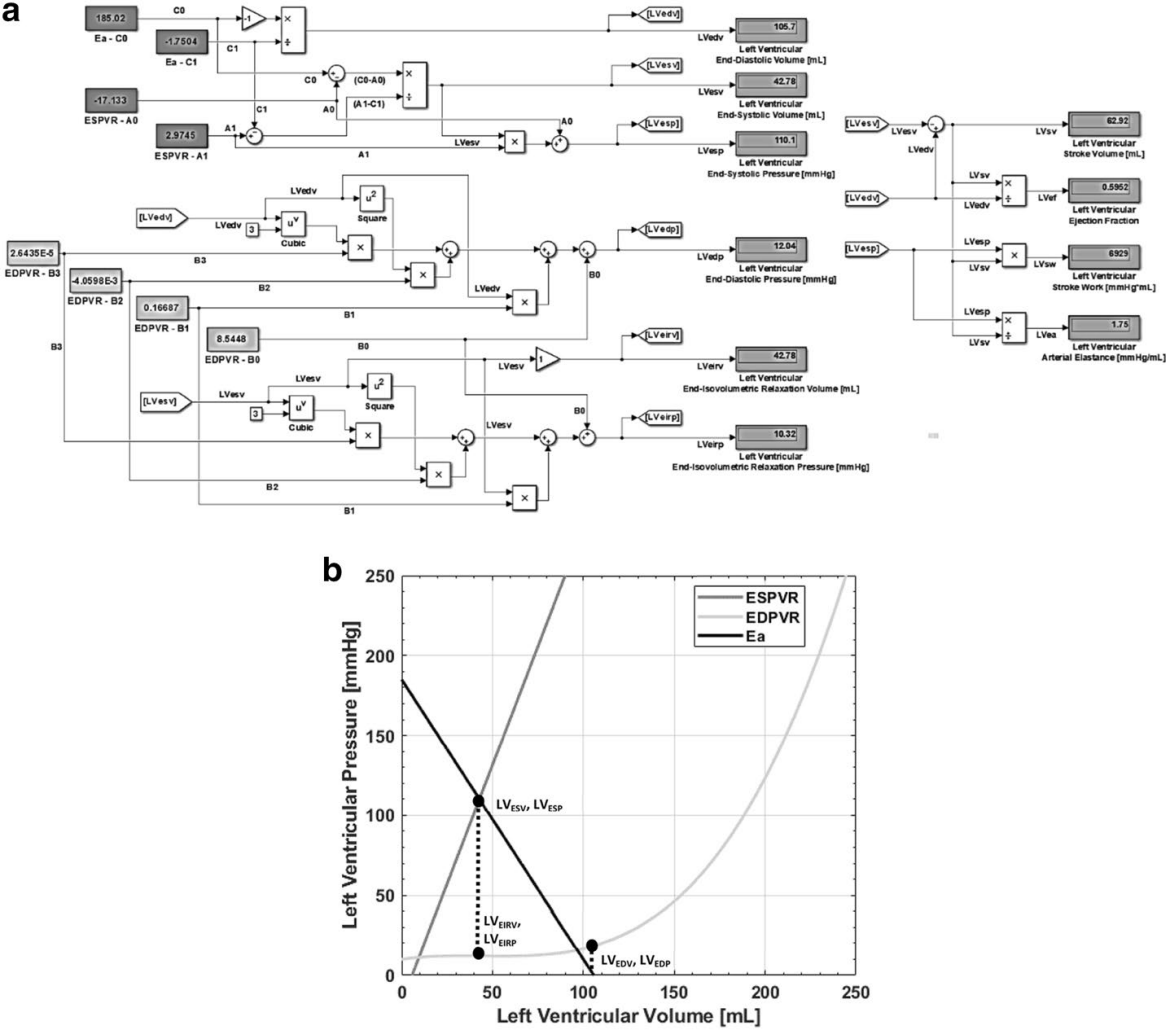
Computational model of example PV loop developed in Simulink™ to reflect Eqs. 4, 5, 6, 7, and 8, to be utilized to find the critical points which define the initiation of phases 1, 2, and 4. **a** reflects the system of equations in this example, capable of solving in real-time. **b** presents a graph of these equations with critical points annotated. The driving values can be interchanged with clinical values for individualized PV assessment, as well as can be controlled over time for determining the effects of preload, afterload, and contractility changes. These values are presented in Tables 1 and 2

Figure 2 presents the computational model and example developed in Simulink™ to reflect Eq. 4 through 9; utilized to find the critical points which define the initiation of each phase. Figure 2a reflects the system of equations in this example, capable of being solved in real-time. Figure 2b presents a graph of these equations, with critical points noted. For this example, based on data collected using DataThief on loop 1 of Fig. 1b: a1 = 2.9745, a0 = − 17.133, b3 = 2.6435E−5, b2 = − 4.0598E−3, b1 = 0.16687, b0 = 8.5448, c1 = − 1.7504, and c0 = 185.02. The computational system produces LV_EDP_ = 12.043 mmHg, LV_EDV_ = 105.71 mL, LV_ESP_ = 110.13 mmHg, LV_ESV_ = 42.785 mL, LV_EIRP_ = 10.323 mmHg, and LV_EIRV_ = 42.785 mL. Using these parameters, LV Stroke Volume (LV_SV_) = 62.93 mL, LV Ejection Fraction (LV_EF_) = 0.595, LV Stroke Work (LV_SW_) = 6929.9 mmHg*mL. These values are presented in Tables 1 and 2. These coefficient values can be interchanged with clinical values for individualized PV assessment, and can be controlled over time for determining the effects of ventricular functional shifts. Utilizing DataThief [48], an open-source program used to extract data from images, these coefficients can be obtained from a plot of a patient’s left ventricular pressure–volume analysis of preload change.

**Table 1:** Input parameters for all simulations presented

	Original	Preload only	Afterload only	Contractility only	Control	HFNEF
Simulation time [s]	10	10	10	10	10	10
Isovolumetric rate [N*sample/s]	225	225	225	225	500	500
Isovolumetric Contraction offset [mL]	1	1	1	1	1	1
Systolic ejection rate [N*sample/s]	2	2	2	2	9	9
Systolic ejection offset [mmHg]	7	7	7	7	20	20
Resistance	0.03	0.03	0.03	0.03	0.05	0.045
Fluid chamber capacity [mL]	517.15	517.15	517.15	517.15	517.15	517.15
Preload pressure [psi]	0.01	0.01	0.01	0.01	0.01	0.01
Pressure full capacity [psi]	10	10	10	10	6	6.6667
Initial volume of fluid [mL]	110.13	110.13	110.13	110.13	210.667	210.48
A1				[2.9745		
				2.9745		
				2.7245		
				2.4745		
	2.9745	2.9745	2.9745	2.2245	1.2407	0.99741
				1.9745		
				1.7245		
				1.4745		
				1.2245]		
A0	− 17.133	− 17.133	− 17.133	− 17.133	33.857	72.586
B3	2.6435E−05	2.6435E−05	2.6435E−05	2.6435E−05	2.6928E−07	1.4046E−05
B2	− 4.0598E−03	− 4.0598E−03	− 4.0598E−03	− 4.0598E−03	− 9.3013E−06	− 2.5351E−03
B1	0.16687	0.16687	0.16687	0.16687	0.026968	0.15836
B0	8.5448	8.5448	8.5448	8.5448	2.9515	− 0.010234
C1			[− 1.7504		[− 1.1365	[− 1.4054
			− 1.7504		− 1.1365	− 1.4054
			− 1.60848		− 1.1365	− 1.4054
	− 1.7504	− 1.7504	− 1.46656	− 1.7504	− 1.1992	− 1.3994
			− 1.32464		− 1.2619	− 1.3934
			− 1.18272		− 1.3247	− 1.3874
			− 1.0408]		− 1.3874	− 1.3814
					− 1.4501]	− 1.3754]
C0		[185.02	[185.02		[211.17	[235.76
		185.02	185.02		211.17	235.76
		190.02	170.02		211.17	235.76
		195.02	155.02		200.96	220.7
	185.02	200.02	140.02	185.02	190.75	205.63
		205.02	125.0		180.53	190.56
		210.02	110.02]		170.32	175.5
		215.02]			160.11]	160.43]

**Table 2:** Results for all simulations presented. Note, error was calculated as the absolute value of the difference between the desired and simulated LV_ESP_, LV_ESV_, LV_EDP_, and LV_EDV_

	Original	Preload only	Afterload only	Contractility only	Control	HFNEF
LV_ESP,i_ [mmHg]	110.13	110.13	110.13	110.13	126.4	140.32
LV_ESV,i_ [mL]	42.785	42.785	42.785	42.785	74.589	67.91
LV_EDP,i_ [mmHg]	12.043	12.043	12.043	12.043	9.3686	21.522
LV_EDV,i_ [mL]	105.71	105.71	105.71	105.71	185.81	167.75
LV_EIRP,i_ [mmHg]	10.323	10.323	10.323	10.323	5.023	3.4517
LV_EIRV,i_ [mL]	42.785	42.785	42.785	42.785	74.589	67.91
LV_SV,i_ [mL]	62.93	62.93	62.93	62.93	111.22	99.84
LV_EF,i_	0.595	0.595	0.595	0.595	0.599	0.595
LV_SW,i_ [mmHg*mL]	6929.9	6929.9	6929.9	6929.9	14058.3	14009.5
Simulated LV_ESP,i_ [mmHg]	110.05	110.05	110.05	110.05	125.8	140.1
Error LV_ESP,i_ [mmHg]	0.08	0.08	0.08	0.08	0.6	0.22
Simulated LV_ESV,i_ [mL]	42.82	42.82	42.82	42.82	73.97	67.19
Error LV_ESV,i_ [mL]	0.035	0.035	0.035	0.035	0.619	0.72
Simulated LV_EDP,i_ [mmHg]	12.71	12.71	12.71	12.71	9.87	22.23
Error LV_EDP,i_ [mmHg]	0.667	0.667	0.667	0.667	0.5014	0.708
Simulated LV_EDV,i_ [mL]	106	106	106	106	185.8	168
Error LV_EDV,i_ [mL]	0.29	0.29	0.29	0.29	0.01	0.25
LV_ESP,f_ [mmHg]	110.13	129.02	77.061	66.075	92.071	109.51
LV_ESV,f_ [mL]	42.785	49.134	31.667	67.953	46.92	37.021
LV_EDP,f_ [mmHg]	12.043	16.783	12.044	12.043	6.1782	6.2607
LV_EDV,f_ [mL]	105.71	122.84	105.71	105.702	110.41	116.64
LV_EIRP,f_ [mmHg]	10.323	10.079	10.597	9.432	4.2242	3.0906
LV_EIRV,f_ [mL]	42.785	49.134	31.667	67.953	46.92	37.021
LV_SV,f_ [mL]	62.93	73.71	74.04	37.75	63.49	79.62
LV_EF,f_	0.595	0.600	0.700	0.357	0.575	0.683
LV_SW,f_ [mmHg*mL]	6929.9	9509.5	5705.8	2494.3	5845.6	8719.1
Simulated LV_ESP,f_ [mmHg]	109.9	128.7	77.84	66.74	92.73	109.9
Error LV_ESP,f_ [mmHg]	0.23	0.32	0.779	0.665	0.659	0.39
Simulated LV_ESV,f_ [mL]	42.95	49.19	31.9	68.18	46.65	37.16
Error LV_ESV,f_ [mL]	0.165	0.056	0.233	0.227	0.27	0.139
Simulated LV_EDP,f_ [mmHg]	12.73	17.21	12.75	12.75	6.711	6.624
Error LV_EDP,f_ [mmHg]	0.687	0.427	0.706	0.707	0.5328	0.3633
Simulated LV_EDV,f_ [mL]	106.1	123.3	106.2	106.2	110.6	116.7
Error LV_EDV,f_ [mL]	0.39	0.46	0.49	0.498	0.19	0.06
ΔLV_SV_ [mL]	0.00	10.78	11.12	− 25.18	− 47.73	− 20.22
ΔLV_EF_	0.000	0.005	0.105	− 0.238	− 0.024	0.087
ΔLV_SW_ [mmHg*mL]	0.0	2579.6	− 1224.1	− 4435.7	− 8212.7	− 5290.5
Initial Heart Rate [bpm]	59.36	59.36	59.36	59.36	59.71	63.54
Final Heart Rate [bpm]	59.94	54.52	43.73	79.69	89.43	70.7
Mean Heart Rate [bpm]	59.9	57.62	49.28	65.04	73.7	65.36

### PV loop modeling utilizing a state machine control architecture approach

**Fig. 3:**
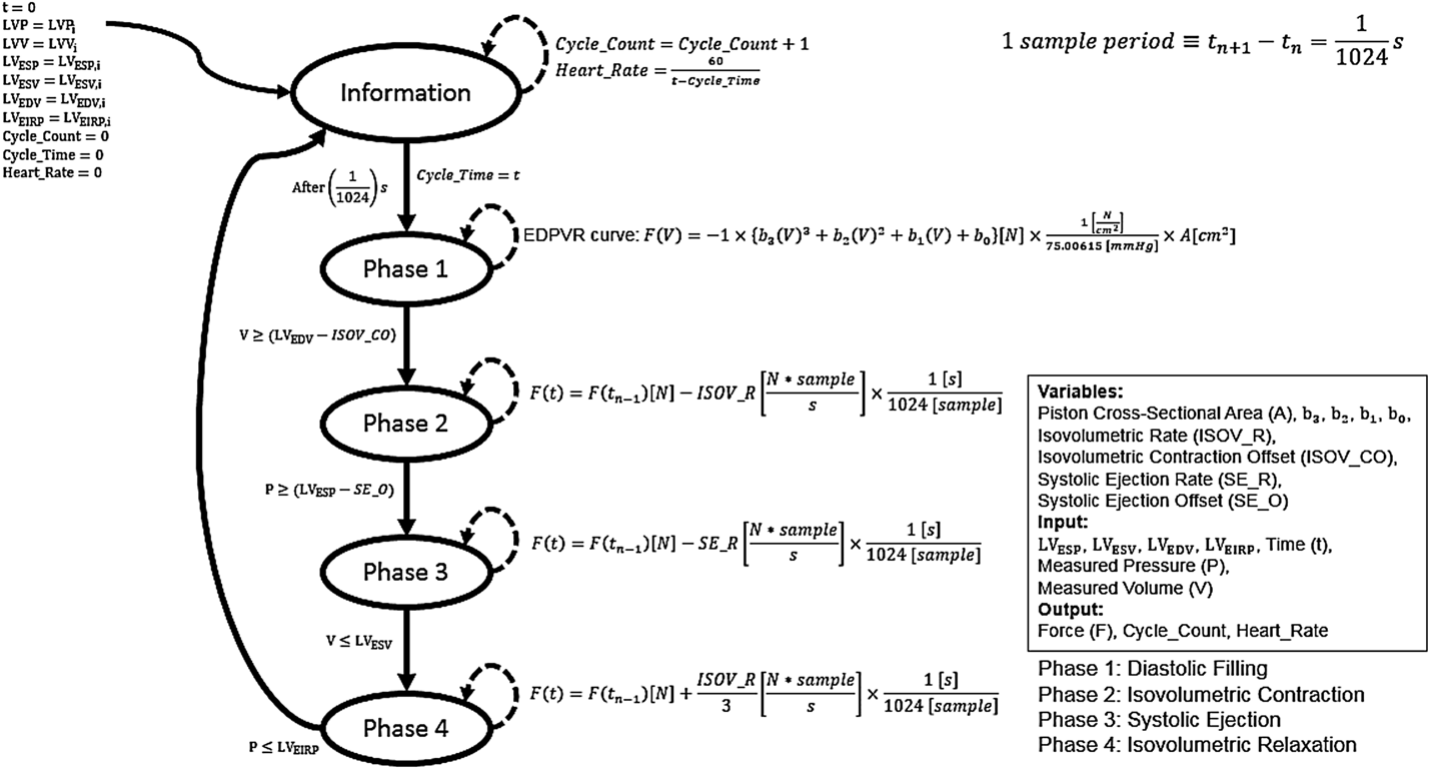
State transition diagram of sequential decision-based control logic represented in Mealy machine structure form was developed to control the transition between left ventricular PV phases. The Variables, parameters that are held constant, are Piston cross-sectional area (A), b_3_, b_2_, b_1_, b_0_, Isovolumetric Contraction Offset, Systolic Ejection Rate, and Systolic Ejection Offset. The Inputs, parameters that can change with time, are ${\text{LV}}_{\text{ESP}}$, ${\text{LV}}_{\text{ESV}}$, ${\text{LV}}_{\text{EDV}}$, ${\text{LV}}_{\text{EIRP}}$, Time (t), Measured Pressure (P), and Measured Volume (V). The Output, the output variable of the model, is Force (F) applied to the piston in Newtons. The single curved arrow donates the initial time-dependent conditions of the model. The oval shapes are the five states of the model. The dotted hoop arrow denotes the output of the state until a specific condition is met. The straight arrows are the transition direction once the condition annotated is satisfied. The sample rate is 1024 Hz

Utilizing Simulink™ Stateflow^®^, a sequential decision-based control logic represented in Mealy machine structure form was developed to control the transition between LV-PV phases. A Mealy machine is appropriate because this application requires that the output values are determined by both its current state and the current input values. A state transition diagram is presented in Fig. 3. The Variables in the block are parameters that are held constant: Piston cross-sectional area (A), b3, b2, b1, b0, Isovolumetric Rate, Isovolumetric Contraction Offset, Systolic Ejection Rate, and Systolic Ejection Offset. The Inputs are parameters that can change with time and are LV_ESP_, LV_ESV_, LV_EDV_, LV_EIRP_, time (t), simulated pressure (P), and simulated volume (V). The Output is the output variables of the model, which is Force (F) applied to the piston in Newtons, Cycle_Count, and Heart_Rate [bpm]. The organization of the state transition diagram follows FSM convention: the single curved arrow donates the initial time-dependent conditions of the model, the oval shapes are the states of the model, the dotted hoop arrows denote the output of the state until a specific condition is met, and the straight arrows are the transition direction once the condition annotated is satisfied. Time (t) is an input variable that discretely changes at the Fundamental Sampling Time of the simulation, $\frac{1}{1024}{\text{s}}$. Correspondingly, the FSM operates at a sampling rate of 1024 Hz. After every complete cycle, the output variables Cycle_Count and Heart_Rate are calculated. Heart rate is determined based on the Cycle_Time that is updated with the current time at the initiation of Phase 1 for every cycle. Isovolumetric Rate is defined as the rate of change in the output variable, F, during isovolumetric relaxation and contraction. For isovolumetric relaxation, this rate is one-third the magnitude when compared to isovolumetric contraction. The Isovolumetric Contraction Offset is defined as the value subtracted from the LV_EDV_ to start the initialization of the Phase 2 state to compensate for the radius of curvature created due to transitioning from fill to eject, as well as the means by which end-diastolic pressure and volume are clinically quantified. The Systolic Ejection Rate is defined as the rate of change in the output variable, F, during systolic ejection. Systolic Ejection Offset is defined as the value subtracted from the LV_ESP_ to start the initialization of the Phase 3 state, establishing LV_EICP_.

### Hydraulic testing model utilizing MathWorks’ Simulink^®^ and Simscape™ toolbox

**Fig. 4:**
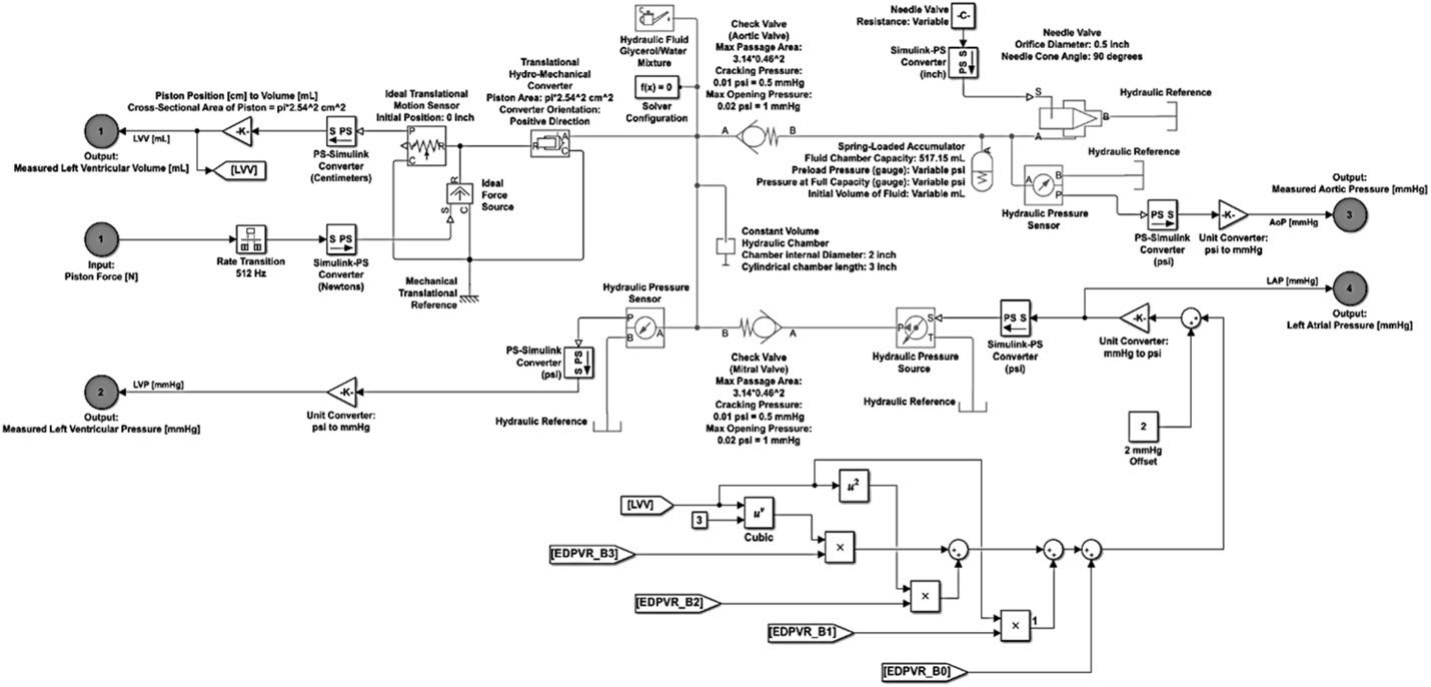
Presented is the hydraulic testing model developed utilizing Simulink^®^ and Simscape™. This system was designed to replicate the dynamics of a force-based piston pump model that drives the pressure within a chamber between two opposing check valves, conditions reflected within the left ventricular portion of an MCS. All block element parameter values that were modified are noted in the diagram, while any parameters not noted were left standard to the block’s original parameter values. Additionally, for any element parameter denoted as ‘Variable’, these values were not left constant for all simulations presented. The hydraulic testing model is a one-input, four-output system. The input is the force [N] applied to the piston and is regulated by means of the Stateflow^®^ control architecture. The outputs are simulated LVV [mL], simulated LVP [mmHg], simulated AoP [mmHg], and LAP [mmHg]

A hydraulic testing model was developed for simulating hydraulic performance as presented in Fig. 4. This system was designed to replicate the dynamics of a force-based piston pump model that drives the pressure within a chamber between two opposing check valves. This constitutes similar conditions observed within the left ventricular portion of an MCS. The Simulink^®^ and Simscape™ block library provided all the necessary components needed to create a hydraulic testing platform capable of simulating this application. All modified parameter values are noted in the diagram, while any parameters not noted were left standard to the block’s original parameter values. Additionally, for any element parameter denoted as ‘Variable’, these values were not left constant for all simulations presented. The values utilized in each simulation, not explicitly declared in Fig. 4, are displayed in Table 1.

The hydraulic testing model is a one-input, four-output system. The input is the force [N] applied to the piston and is regulated by means of the Stateflow^®^ control architecture. The outputs are simulated left ventricular volume (LVV) [mL], simulated left ventricular pressure (LVP) [mmHg], simulated aortic pressure (AoP) [mmHg], and left atrial pressure (LAP) [mmHg]. LVP and LVV are utilized by the Stateflow^®^ control logic to govern state transitions while AoP and LAP are used for system fidelity and plotting purposes. The input force is applied to the Ideal Force Source block element which is then directed to an Ideal Translational Motion Sensor which converts an across variable measured between two mechanical translational nodes into a control signal proportional to position. The position signal is then converted into volume [mL] based on a piston diameter of 2 inches, thus a cross-sectional area of π × 2.54^2^ = 20.27 cm^2^. The input force [N] is also applied to a Translational Hydro-Mechanical Converter which converts hydraulic energy into mechanical energy in the form of translational motion of the converter output member. Two check valves (aortic and mitral), positioned in opposing directions, regulate the fluid flow direction as seen in the left ventricular section of an MCS. A Constant Volume element is positioned between the two check valves to simulate a constant volume filling chamber. A Hydraulic Pressure Sensor is positioned between the opposing check valves to monitor LVP, then outputs the simulated values to the Stateflow^®^ control logic.

Upstream to the mitral valve is a Hydraulic Reference source block governed by the EDPVR curve function with respect to simulated volume, LVV, and increased by an offset of 2 mmHg to ensure proper flow through the mitral check valve. This establishes a dynamic LAP, the initial pressure condition of the left heart. LAP is outputted from the model here for plotting purposes. Downstream to the aortic valve is a Spring-Loaded Accumulator block. This block element consists of a preloaded spring and a fluid chamber. As the fluid pressure at the inlet of the accumulator becomes greater than the prescribed preload pressure, fluid enters the accumulator and compresses the spring, creating stored hydraulic energy. A decrease in the fluid pressure causes the spring to decompress and eject the stored fluid into the system. The spring motion is restricted by a hard stop when the fluid volume becomes zero, as well as when the fluid volume is at the prescribed capacity of the fluid chamber. These settings are utilized to regulate the compliance,$\frac{{\Delta {\text{V}}}}{{\Delta {\text{P}}}}$, of the aorta. Immediately following is Hydraulic Pressure Sensor measuring AoP.

Additionally, a needle valve was positioned downstream to the aortic valve to simulate the resistance to flow contributed to the branching arteries of the aortic arch, as well as provide the capability to simulate the effects of increasing and decreasing resistance with time. As was previously stated, all block element parameter values that were modified are noted in the diagram presented in Fig. 4, while any parameters not noted were left standard to the block’s original parameter values. For any element parameter denoted as ‘Variable’, these values were not left constant for all simulations presented. For each simulation, these values are displayed in Table 1.

## Results

The computational model effectively executed the trials assessing the performance of the FSM architecture. Solver settings and simulated fluid type were held constant through the analysis. The results presented were produced with the MathWorks’ ode14x (fixed-step, extrapolation) using a fundamental sampling time of $\frac{1}{1024}$ s. This solver was chosen to accelerate the simulations and ensure the resultant model is compatible with deterministic hardware systems. Validation of this solver was performed against a variable-step variable-order solver (ODE15 s) to ensure accuracy. The fluid selected is a glycerol/water mixture with a fluid density of 1107.1 kg/m^3^ and a kinematic viscosity of 3.3 centistoke [49]. These characteristics equate to a fluid temperature of 25 °C or 77 °F.

The input variables utilized for each presented simulation are displayed in Table 1, while the results of each simulation are displayed in Table 2. All simulations were performed utilizing discrete changes, evenly incremented between the designated initial and final LV_ESP_, LV_ESV_, LV_EDP_, and LV_EDV_ over a 10 s total simulation time. Each discrete variable is controlled by means of a Lookup Table element block that outputs the modified variable value, dependent on the specific cycle count number. Note, any variable presented as a vector, changes with each cycle count, i.e. $[ 1,{ 2},{ 3}, \cdots ,{\text{n}}]$ where the nth value represents the input variable value for the entirety of the corresponding cycle. If a simulation has more cycles than input vector elements, then the system continues with a zero-order hold of the last value.

The parameters for the Spring-Loaded Accumulator block were developed based on a desired LVP response due to aortic compliance. The desired response consisted of a physiological correct AoP waveform and a peak-to-peak AoP amplitude of approximately 40 mmHg, corresponding to a normal range of 120/80. The baseline of this response was created at a heart rate of 60 bpm and a compliance of 1. This corresponded to an Isovolumetric Rate of 225 N*sample/s, a Resistance value of 0.03, a Fluid Chamber Capacity of 517.15 mmHg, a Preload Pressure of 0.01 psi, and a Pressure at Full Capacity of 10.01 psi. Given the relationship $\frac{1}{R*C} = I$, where R is resistance, C is compliance, and I is the Impedance, I was held constant for all simulations using I = 33.333. For the simulations that required a heart rate beyond 60 bpm, the Isovolumetric Rate had to be consequently increased. Utilizing this relationship to sustain a peak-to-peak AoP amplitude of 40 mmHg, the Fluid Chamber Capacity and the Preload Pressure was held constant, while Resistance and Pressure at Full Capacity was modified to produce the desired heart rate while sustaining aortic performance. Lastly, the Initial Volume of Fluid for each simulation was calculated to create an initial LVP corresponding to LV_ESP_. This was done to decrease the amount of initial cycles necessary to achieve simulation stability to 1. All values utilized for these parameters are presented in Table 1. Error was calculated as the absolute value of the difference between the desired and simulated LV_ESP_, LV_ESV_, LV_EDP_, and LV_EDV_.

A LV-PV loop; LVP, LAP, and AoP versus time; and volume versus time graphs for the 10 s total simulation time was presented for each simulation. Note, the driving force [N] produced by the FSM can be derived from the presented LVP and LVV plots by means of ${\text{Force }}\left[ {\text{N}} \right] = {\text{Pressure }}\left[ {\text{mmHg}} \right] \times \left[ {1\frac{\text{N}}{{{\text{cm}}^{2} }}/75.00615 {\text{mmHg}}} \right] \times {\text{Piston area }}\left[ {{\text{cm}}^{2} } \right]$ . The piston cross-sectional area is π × 2.54^2^ = 20.27 cm^2^. The piston position [cm] can additionally be derived from the volume time plot by means of ${\text{Piston position }}\left[ {\text{cm}} \right] = {\text{Volume }}\left[ {{\text{cm}}^{ 3} } \right] \div {\text{Piston area }}\left[ {{\text{cm}}^{2} } \right]$.

### Computational model verification

**Fig. 5:**
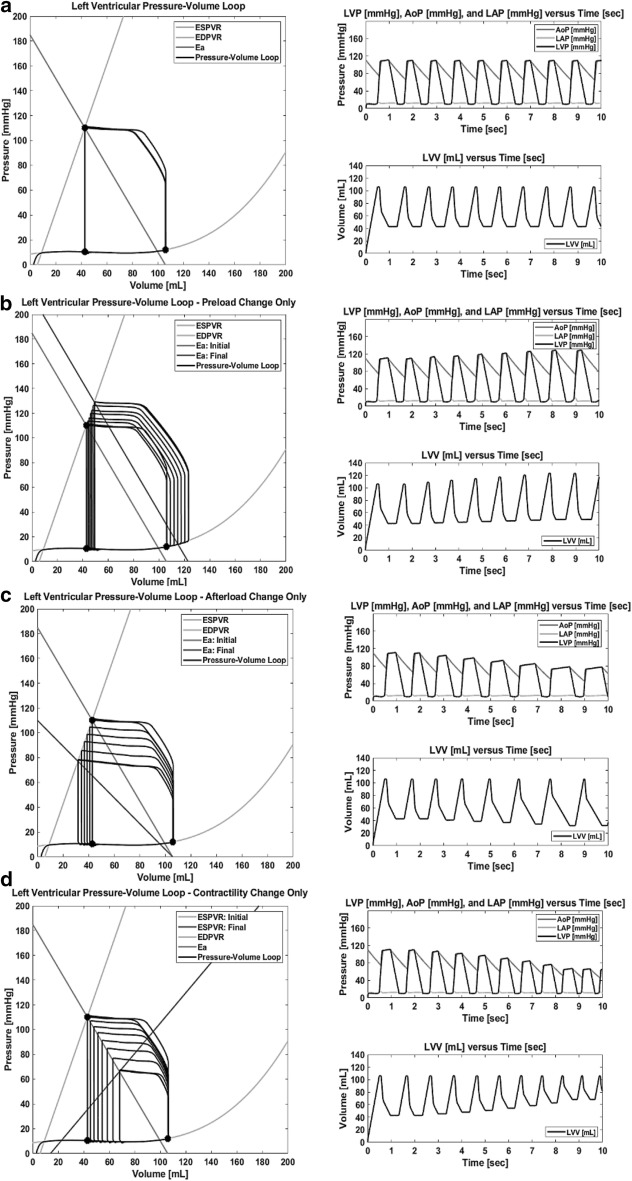
The outlined approach was effective at simulating the characteristic LV-PV relationship. Preload, afterload, and contractility changes in time were simulated by means of manipulating the input variables of the computational model via evenly-spaced discrete increments that change per cycle count. The LV-PV loop, pressure versus time, and volume versus time graphs are presented for each simulation. Displayed in **a** is the derived LV-PV loop, based on the computational model parameters determined using DataThief on loop 1 of Fig. 1b and presented in Fig. 2. The parameters for this LV-PV loop constitutes the initial conditions for the subsequent simulations. **b** presents the system correctly responding to a discrete change in preload. **c** reveals the correct afterload change response to the PV relationship. **d** displays the correct system response to contractility change. Each simulation was run for a total simulation time of 10 s and the system takes one cycle before it settles. The system functions consistently for every preceding cycle. The heart rate begins at approximately 60 bpm for each simulation. The reference force [N] produced by the FSM as well as the piston position [cm] can be derived from these time graphs

The LV-PV loop critical point computational model and FSM approach was effective at driving the hydraulic testing model to produce the characteristic LV-PV relationship as presented in Fig. 5. The computational model parameters are the same as those presented in Fig. 2. As can be shown from the graph, with known ESPVR, EDPVR, and E_a_ curves, the computational model successfully provided the correct LV_ESP_, LV_ESV_, LV_EDP_, LV_EDV_, LV_EIRP_, and LV_EIRV_ transition points within the state transition logic to produce the prescribed LV-PV relationship. Table 1 contains all input parameters and Table 2 presents the results of all simulations performed. For each LV-PV loop graph, the initial LV end-systolic and end-diastolic datasets are denoted with circle points. Figure 5a displays the LV-PV loop based on data collected using DataThief on loop 1 of Fig. 1b. The results presented reveal an error between the desired and simulated end-systolic and end-diastolic transition points in the datasets of less than 1 mmHg and 1 mL, respectively.

The system takes one cycle to initialize from a rest state before the control topology functions consistently for the remainder of the simulation. Additionally, the isovolumetric and systolic offsets and rates, necessary to achieve this response are noted in Table 1. Figure 5a also presents the LVP, LAP, and AoP versus time and volume versus time graphs for the 10 s total simulation time. The reference force [N] produced by the FSM as well as the piston position [cm] can be derived from these time graphs.

### Preload, afterload, and contractility changes in time

As presented in Fig. 5b–d, the outlined approach was effective at simulating preload, afterload, and contractility changes in time by means of discretely manipulating the computational model over time. The initial parameters of the computational model are the same as those presented in Fig. 5a and presented in Table 1. Presented for each simulation is the LV-PV loop; LVP, LAP, and AoP versus time; and volume versus time graphs for the 10 s total simulation time.

As shown in Fig. 5b, the system displays the correct preload change response to the PV relationship as displayed in Fig. 1b. The E_a_ was initially defined by the equation ${\text{P}} = - 1.7504\left( {\text{V}} \right) + 185.02$. The y-axis intercept was increased from 185.02 mmHg at a rate of 5 mmHg per cycle, ending with a y-axis intercept of 215.02 mmHg for the last completed cycle. The results report an error of less than 1 mmHg and 1 mL for all targeted pressures and volumes.

Presented in Fig. 5c, the system reveals the correct afterload change response to the PV relationship as shown in Fig. 1c. E_a_ is initially defined by the equation ${\text{P}} = - 1.7504\left( {\text{V}} \right) + 185.02$. The y-axis intercept was decreased from 185.02 mmHg at a rate of 15 mmHg per cycle, ending with a y-axis intercept of 110.02 mmHg for the last completed cycle. The slope of the E_a_ was decreased from − 1.7504 mmHg/mL concluding with a slope of − 1.0408 mmHg/mL. This rate of change for the E_a_ slope was derived from the 15 mmHg per cycle y-axis rate of increase to achieve a consistent x-intercept, as shown in Fig. 1c. The results indicate an error of less than 1 mmHg and 1 mL for all targeted datasets.

As presented in Fig. 5d, the system displays the correct contractility change response to the PV relationship as revealed in Fig. 1d. The ESPVR curve is initially defined by the equation ${\text{P}} = 2.9745\left( {\text{V}} \right) - 17.133$. The slope of the ESPVR curve was decreased from 2.9745 mmHg/mL, concluding with a slope of 1.2245 mmHg/mL for the last completed cycle. The results report an error of less than 1 mmHg and 1 mL for all targeted pressures and volumes.

### Clinical assessment of outlined approach

**Fig. 6:**
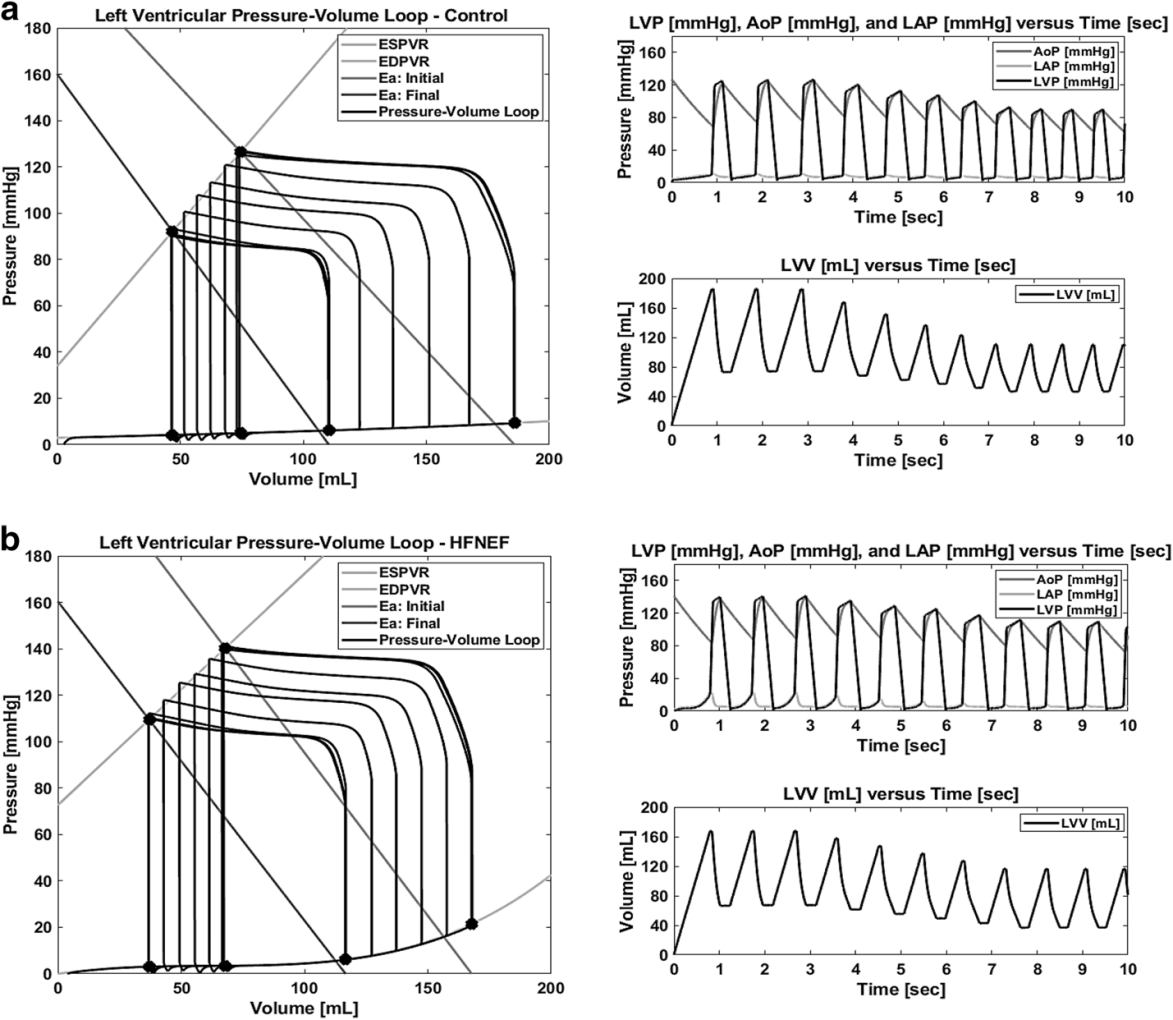
The outlined approach was effective at simulating Heart Failure with Normal Ejection Fraction (HFNEF) and the Control developed by means of a preload reduction analysis conducted in 2008 by Westermann et al. [50] and presented in Fig. 1 of their investigation. The ESPVR, E_a_, and EDPVR curve coefficients were developed utilizing DataThief to find the associated LV_ESP_, LV_ESV_, LV_EDP_, and LV_EDV_ for the initial and final loops, as well as evaluate the EDPVR curve. These datasets were analyzed over a 10 s total simulation time and for each simulation is the LV-PV loop; LVP, LAP, and AoP versus time; and volume versus time graphs. **a** presents the Control where the slope and y-intercept of E_a_ was divided into evenly-spaced increments to constitute 4 intermediate discrete steps between the initial and final cycle parameters. HFNEF is presented in **b**. The slope and y-intercept of E_a_ was also divided into evenly-spaced increments to constitute 4 intermediate discrete steps between the initial and final cycle parameters. For both simulations, the results produced an error of less than 1 mmHg and 1 mL for all targeted datasets and reflect a mean heart rate [bpm] within the range of mean values noted in the reference material. The reference force [N] produced by the FSM as well as the piston position [cm] can be derived from these time graphs

Figure 6 displays the results of simulating Heart Failure with Normal Ejection Fraction (HFNEF) and the Control developed by means of a preload reduction analysis conducted in 2008 by Westermann et al. [50] and presented in Fig. 1 of their investigation. The ESPVR, E_a_, and EDPVR curve coefficients were developed utilizing DataThief to find the associated LVESP, LV_ESV_, LV_EDP_, and LV_EDV_ for the initial and final loops, as well as evaluate the EDPVR curve. These datasets were analyzed over a 10 s total simulation time and for each simulation are the LV-PV loop; LVP, LAP, and AoP versus time; and volume versus time graphs. Both simulations reflect a mean heart rate [bpm] within the range of mean values noted in the reference material. All parameter values are presented in Table 1 and the results are in Table 2.

The Control is presented in Fig. 6a. The ESPVR curve was found to be defined by the equation ${\text{P}} = 1.2407\left( {\text{V}} \right) + 33.857$ and the EDPVR curve was found to be ${\text{P}} = 2.6928{\text{E}} - 7\left( V \right)^{3} + - 9.3013{\text{E}} - 6\left( V \right)^{2} + 0.026968\left( V \right) + 2.9515$. E_a_ is initially defined by the equation ${\text{P}} = - 1.1365\left( {\text{V}} \right) + 211.17$ and defined by the equation ${\text{P}} = - 1.4501\left( {\text{V}} \right) + 160.11$ for the final cycle. The slope and y-intercept of E_a_ was divided into evenly-spaced increments to constitute 4 intermediate discrete steps between the initial and final cycle parameters. The results indicate an error of less than 1 mmHg and 1 mL for all targeted datasets.

HFNEF is presented in Fig. 6b. The ESPVR curve was found to be ${\text{P}} = 0.99741\left( {\text{V}} \right) + 72.586$ and the EDPVR curve was found to be ${\text{P}} = 1.4046{\text{E}} - 5\left( V \right)^{3} + - 2.5351{\text{E}} - 3\left( V \right)^{2} + 0.15836\left( V \right) + - 0.010234$. E_a_ is initially defined by the equation ${\text{P}} = - 1.4054\left( {\text{V}} \right) + 235.76$ and defined by the equation ${\text{P}} = - 1.3754\left( {\text{V}} \right) + 160.43$ for the final cycle. The slope and y-intercept of E_a_ was divided into evenly-spaced increments to constitute 4 intermediate discrete steps between the initial and final cycle parameters. The results produced an error of less than 1 mmHg and 1 mL for all targeted datasets.

## Discussion

A novel method for simulating LV-PV control functionality utilizing explicitly defined preload, afterload, and contractility was delivered for cardiovascular intervention assessment. The proposed aggregate model consists of three subsystems which include a preload, afterload, and contractility sensitive computational setpoint calculator (“PV loop critical point determination” section), a FSM controller (“PV loop modeling utilizing a state machine control architecture approach” section), and a hydraulic testing system (“Hydraulic testing model utilizing MathWorks’ Simulink® and SimscapeTM toolbox” section). The computation model provides pressure and volume setpoints based on the coefficients revealed by best fit equations for ESPVR, EDPVR, and E_a_. The acquired setpoints drive the FSM controller to perform the prescribed PV relationship. Then the hydraulic testing system, which reproduces conditions comparable to those found in a left heart MCS with cardiac piston actuation, simulates the PV relationship defined by the inputs to the computational model.

The resulting solution was capable of being compiled for hardware control in a MCS through the architecture and solver type employed; deterministic processing is achievable and runtime setpoint changes can be made. Simulink^®^ and its supplemental product library was effective at developing reproducible clinical conditions, which would be determined through an individualized clinical PV analysis, simulated in silico for this work with ability to translate to future in vitro investigations. This provides an MCS with the capabilities to investigate the pathophysiology for a specific individual, with or without VAD support, by reproducing the precise PV relationship defined by their left ventricular functionality.

In silico verification of the LV-PV loop critical point computational model, FSM control architecture, and hydraulic testing system support this modeling approach as an effective means of simulating the LV-PV relationship. In this work, a novel method for simulating the characteristic EDPVR curve and LAP during diastolic filling was presented. This approach proved to be an effective means of capturing the nuisances in those sections of the PV curve that are critical for diastolic operation of mechanical circulatory support systems and not found in prior computational models [15, 41].

As shown in Fig. 5a and Table 2, the computational model was able to create specific points that the FSM was able to utilize as features governing the transition between LV-PV states, given a clinical preload analysis, similar to Fig. 1b. Additionally, the hydraulic testing model was able to produce a suitable degree of realism to be able to evaluate the feasibility of this methodology, producing realistic conditions to include LAP and AoP. The delivered capabilities enable control of the PV relationship beyond that presented in prior work on elastance based control with respect to dynamic afterload response [21, 24] and software-oriented control [44].

A key result of this investigation is a novel in silico method for simulating LV-PV relationships based on an analysis of a patient’s ESPVR, EDPVR, and E_a_ curves. Displayed in Fig. 6 is the characteristic LV-PV loop of two individuals presented in the research conducted by Westermann et al. [50]. Simulated is Heart Failure with Normal Ejection Fraction (HFNEF) and the Control developed by means of a preload reduction analysis and quantified by means of data capture tools. Both simulations reflect a mean heart rate [bpm] within the range of mean values described in the reference material. This capability enables the utilization of the breadth of published PV curves on various patient types in the literature; illustrating how the digitized data from these graphs can be utilized with the computational model presented. Additionally, this FSM model could be implemented in embedded physiological control applications that are utilizing model predictive control and require a computationally efficient left ventricular simulator.

## Conclusion

The limitations of this approach are mainly the ideal hydraulic testing system and use of anticipatory limits in transition points of the PV loop. If a force is exerted into this computational model of the hydraulic system, the system responds with the corresponding pressure instantaneously within that sample period. There was no modeled delay or rise time in the actuation components. This consideration is made in the FSM by increasing force incrementally instead of applying a constant desired force. Some parameters which define the hydraulic system, such as the parameters within the Spring-Loaded Accumulator are ideal assumptions based off a desired performance of the system. The focus of this work was on the control architecture that can be adjusted to a variety of hardware platforms through manipulation of the output signal magnitude and response characteristics. Additionally, pressure sensor feedback is ideal using this modeling approach. The sensor sampling rate was set to 512 Hz and assumed an ideal sensor with low noise. Additionally, a manual offset was made to the transition from diastolic filling to isovolumetric contraction of the system; enabling a ramping from the transition of fill to eject. Moreover, an offset was utilized in the transition from isovolumetric contraction to ejection in order to allow the pressure to slowly increase to the desired LV_ESP_ during ejection.

Future work includes a sensitivity analysis regarding resistance, compliance, and force rates. This analysis will be useful in that it will quantify the exact limitations of the hydraulic testing system as well as the range of accuracy of the FSM approach. Isolated in vitro testing of this approach will be conducted on a nested-loop hydraulic system before being incorporated into an MCS for investigating accurate cardiovascular hemodynamic considerations, such as the accuracy of pressure and flowrate sensor feedback. Additionally, what-if scenarios will be conducted on a MCS in order to create feasible scenarios to which a patient may experience.

This research will assist in producing an investigatory method and MCS control logic that will advance the medical community by improving left ventricular in vitro analysis capabilities. The ability of an MCS to be able to replicate the exact PV relationship that define the pathophysiology allows for a robust in vitro analysis to be completed. This ventricular model for ventricular function could also be coupled with aortic and left atrium computational fluid dynamics (CFD) models that require inlet and outlet conditions manifested by the left ventricle. The FSM approach is computationally efficient due to the explicit computation, and simple transition logic, which is preferential when small time steps and high iteration solvers are being employed. It was this efficiency and portability in the outcome that has made this work impactful for a variety of investigative purposes.

## Abbreviations

AoP [mmHg]: aortic pressure
E_a_: arterial elastance
CFD: computational fluid dynamics
CHF: Congestive Heart Failure
CO: cardiac output
CVD: cardiovascular disease
EDPVR: end-diastolic pressure–volume relationship
ESPVR: end-systolic pressure–volume relationship
FSM: finite state machine
HFNEF: Heart Failure with Normal Ejection Fraction
LAP [mmHg]: left atrial pressure
LV: left ventricular
LVAD: left ventricular assist device
LV_EF_: left ventricular ejection fraction
LV_EDP_ [mmHg]: left ventricular end-diastolic pressure
LV_EDV_ [mmHg]: left ventricular end-diastolic volume
LV_EICP_ [mmHg]: left ventricular end-isovolumetric contraction pressure
LV_EICV_ [mmHg]: left ventricular end-isovolumetric contraction volume
LV_EIRP_ [mmHg]: left ventricular end-isovolumetric relaxation pressure
LV_EIRV_ [mmHg]: left ventricular end-isovolumetric relaxation volume
LV_ESP_ [mmHg]: left ventricular end-systolic pressure
LV_ESV_ [mmHg]: left ventricular end-systolic volume
LVP [mmHg]: left ventricular pressure
LV-PV: left ventricular pressure–volume
LV_SV_ [mL]: left ventricular stroke volume
LV_SW_ [mmHg*mL]: left ventricular stroke work
LVV [mL]: left ventricular volume
MCS: mock circulatory system
PSM: patient-specific modeling
SV: stroke volume
VAD: ventricular assist device
V&V: verification and validation
